# Effects of positive end-expiratory pressure and recruitment maneuvers in a ventilator-induced injury mouse model

**DOI:** 10.1371/journal.pone.0187419

**Published:** 2017-11-07

**Authors:** Laura A. Cagle, Lisa M. Franzi, Angela L. Linderholm, Jerold A. Last, Jason Y. Adams, Richart W. Harper, Nicholas J. Kenyon

**Affiliations:** 1 Center for Comparative Respiratory Biology and Medicine, University of California, Davis, Davis, CA, United States of America; 2 Division of Pulmonary, Critical Care, and Sleep Medicine, School of Medicine, University of California, Davis, Davis, CA, United States of America; National Yang-Ming University, TAIWAN

## Abstract

**Background:**

Positive-pressure mechanical ventilation is an essential therapeutic intervention, yet it causes the clinical syndrome known as ventilator-induced lung injury. Various lung protective mechanical ventilation strategies have attempted to reduce or prevent ventilator-induced lung injury but few modalities have proven effective. A model that isolates the contribution of mechanical ventilation on the development of acute lung injury is needed to better understand biologic mechanisms that lead to ventilator-induced lung injury.

**Objectives:**

To evaluate the effects of positive end-expiratory pressure and recruitment maneuvers in reducing lung injury in a ventilator-induced lung injury murine model in short- and longer-term ventilation.

**Methods:**

5–12 week-old female BALB/c mice (n = 85) were anesthetized, placed on mechanical ventilation for either 2 hrs or 4 hrs with either low tidal volume (8 ml/kg) or high tidal volume (15 ml/kg) with or without positive end-expiratory pressure and recruitment maneuvers.

**Results:**

Alteration of the alveolar-capillary barrier was noted at 2 hrs of high tidal volume ventilation. Standardized histology scores, influx of bronchoalveolar lavage albumin, proinflammatory cytokines, and absolute neutrophils were significantly higher in the high-tidal volume ventilation group at 4 hours of ventilation. Application of positive end-expiratory pressure resulted in significantly decreased standardized histology scores and bronchoalveolar absolute neutrophil counts at low- and high-tidal volume ventilation, respectively. Recruitment maneuvers were essential to maintain pulmonary compliance at both 2 and 4 hrs of ventilation.

**Conclusions:**

Signs of ventilator-induced lung injury are evident soon after high tidal volume ventilation (as early as 2 hours) and lung injury worsens with longer-term ventilation (4 hrs). Application of positive end-expiratory pressure and recruitment maneuvers are protective against worsening VILI across all time points. Dynamic compliance can be used guide the frequency of recruitment maneuvers to help ameloriate ventilator-induced lung injury.

## Introduction

Acute lung injury (ALI) and acute respiratory distress syndrome (ARDS) have a reported mortality rate of 35 to 65% depending on the cohort studied.[1,2] Positive-pressure mechanical ventilation, although life-saving, can worsen lung injury and cause the clinical syndrome known as ventilator-induced lung injury.[3,4] Ventilator-induced lung injury (VILI) results from overdistension of the lung parenchyma known as volutrauma, repetitive collapse and re-expansion of alveoli leading to atelectrauma, or both. This mechanical stretch induces the release of inflammatory mediators, or biotrauma, perpetuating this injury.[5] VILI has received increasing attention because a strategy to limit tidal stretch improved outcomes in ARDS and reduction in tidal volume ventilation resulted in a dramatic improvement in mortality rates in acute lung injury and acute respiratory distress syndrome.[2,6]

Low-tidal volumes and maintenance of positive-end expiratory pressure (PEEP) are the cornerstones of lung-protective ventilation. Lung-protective ventilation focuses on minimizing end-inspiratory stretch (using low tidal volumes) and minimizing derecruitment of the lung (application of PEEP).[4,7] However, even with protective mechanical ventilation recent studies have shown that acute lung injury continues to occur, with a paucity of effective therapeutic modalities.[8] Furthermore, several recent studies have documented that failure to recognize ARDS and implement timely lung protective strategies remains a widespread problem in intensive care units.[9,10] Studies detailing the temporal evolution of ventilator-induced acute lung injury are still lacking in the current literature, and limited clinical studies are conflicting regarding the importance of early institution of low-tidal volume ventilation (LTV) in determining clinical outcomes.[11,12]

Identification of early mechanisms that result in VILI may allow therapeutic interventions aimed at reducing or even preventing lung injury. Interventions targeting PEEP have proven modestly successful in ameliorating some of the injurious effects of ventilation and, in conjunction with recruitment maneuvers (RM), can be protective.[13,14,15] The optimal timing of implementing PEEP and recruitment maneuvers has yet to be evaluated in a translational model. In addition, the fundamental mechanisms (atelectrauma and biotrauma) that lead to VILI are poorly understood.

Mechanical ventilation with high tidal volume (HTV) or high peak inspiratory pressure, pulmonary or systemic administration of endotoxin, or inhalation or instillation of live bacteria are the three most commonly applied models of acute lung injury studied in animals.[16] Mouse studies have focused on supraphysiologic tidal volumes of 20 to 45 ml/kg to stimulate an injurious response with durations of ventilation varying from two to six hours.[17,18,19,20,21,22,23] and lung injury has been shown to occur with short-term ventilation. However, a direct comparison between short-term and long-term ventilation in a translational model is currently lacking in the literature and it is also unknown at what time point ventilation becomes injurious.

The aim of this study was to assess the short-term biophysical changes elicited by low versus high tidal volume ventilation, using tidal volumes that are used clinically, and to assess the effects of PEEP and RM on injury severity in the setting of both low and high tidal volumes. We hypothesized that two to four hours of positive pressure, high tidal volume ventilation–absent a second pulmonary insult–would result in progressive worsening of pulmonary mechanics, lung inflammation, and lung injury. A secondary hypothesis was that the institution of clinically relevant PEEP and RM could ameliorate early ventilator-induced lung injury.

## Methods

### Definitions

For this study, low tidal volume ventilation was defined at a TV 8 ml/kg and high tidal at 15 ml/kg. Addition of positive end-expiratory pressure is equivalent to the use of 4cmH_2_O positiv epressure and zero positive end-expiratory pressure (ZEEP, 0cm H_2_O). Recruitment maneuvers have been defined as 20 cmH_2_O PEEP for 10 seconds applied every 20 minutes. Short- and long-term ventilation were defined as ≤ 2 and 4 hours, respectively. Individual groups have been defined in the Tables 1–4.

**Table 1:** Low and high TV +/- PEEP with RM.

Air control	Low-tidal volume ventilation	High-tidal volume ventilation
*(n = 5)*	Tidal volume 8 ml/kg PEEP 0 cmH_2_O Recruitment maneuvers *(n = 5)*	Tidal volume 15 ml/kg PEEP 0 cmH_2_O Recruitment maneuvers *(n = 5)*
	Tidal volume 8 ml/kg PEEP 4 cmH_2_O Recruitment maneuvers *(n = 5)*	Tidal volume 15 ml/kg PEEP 4 cmH_2_O Recruitment maneuvers *(n = 5)*

**Table 2:** Low and high TV +/- PEEP with or without RM.

Air control	Low-tidal volume ventilation	High-tidal volume ventilation
(n = 5)	Tidal volume 8 ml/kg PEEP 4 cmH_2_O Recruitment maneuvers *(n = 5)*	Tidal volume 15 ml/kg PEEP 0 cmH_2_O Recruitment maneuvers *(n = 5)*
	Tidal volume 8 ml/kg PEEP 4 cmH_2_O No recruitment maneuvers *(n = 5)*	Tidal volume 15 ml/kg PEEP 0 cmH_2_O No recruitment maneuvers *(n = 5)*

**Table 3:** Two hour mechanical ventilation.

Air control	Low-tidal volume ventilation	High-tidal volume ventilation
*(n = 10)*	Tidal volume 8 ml/kg PEEP 4 cmH_2_O Recruitment maneuvers *(n = 10)*	Tidal volume 15 ml/kg PEEP 0 cmH_2_O Recruitment maneuvers *(n = 10)*
	Tidal volume 8 ml/kg PEEP 4 cmH_2_O No recruitment maneuvers *(n = 10)*	Tidal volume 15 ml/kg PEEP 0 cmH_2_O No recruitment maneuvers *(n = 10)*

**Table 4:** Two versus four hour mechanical ventilation.

Air control	Low-tidal volume ventilation	High-tidal volume ventilation
*(n = 5)*	Tidal volume 8 ml/kg PEEP 4 cmH_2_O Recruitment maneuvers 2 hours *(n = 5)*	Tidal volume 15 ml/kg PEEP 0 cmH_2_O Recruitment maneuvers 2 hours *(n = 5)*
	Tidal volume 8 ml/kg PEEP 4 cmH_2_O Recruitment maneuvers 4 hours *(n = 5)*	Tidal volume 15 ml/kg PEEP 0 cmH_2_O Recruitment maneuvers 4 hours *(n = 5)*

### Animals

5–12-week-old, 16–24 gram, female BALB/c mice (n = 85) were obtained from Jackson Laboratory (Bar Harbor, ME) and allowed to acclimatize for one week prior to study enrollment. Animals were housed in plastic cages over autoclaved bedding in a HEPA-filtered laminar flow cage rack on a 12-hour light/dark cycle. All mice were allowed free access to water and a standard diet (Purina Rodent Chow). Procedures with the mice were performed in accordance with an approved IACUC protocol. Mice were routinely screened and cared for by the veterinary staff of the Animal Resource Service at the University of California, Davis in AALAC-accredited facilities.

### Anesthetic protocol

Mice were sedated with dexmedetomidine (200 mcg/kg, IP), tiletamine-zolazepam (40 mg/kg, IP), and buprenorphine (0.1 mg/kg, SC) for mechanical ventilation. Lactated Ringers (400 μl) was given IP prior to anesthesia and every 1 hour during the study period. Dexmedetomidine (100 mcg/kg, IP) and tiletamine-zolazepam (10 mg/kg, IP) were administered at 2 and 3 hours after induction and buprenorphine (0.1 mg/kg, SC) was given at 3 hours after induction for additional sedation or as needed for further sedation. Lidocaine (1%) 20 μl dosage was given SC overlying the trachea along the ventral cervical region prior to tracheostomy. Mice assigned to the air control group were sedated, as described above, but not mechanically ventilated.

### Mechanical ventilation protocol

Mice were placed in dorsal recumbent position and initial heart rate (HR), respiratory rate (RR), and pulse oximetry (SpO_2_) were measured. An incision was made along the ventral cervical region and a cannula (1mm outer diameter, 20mm length; Harvard apparatus, Holliston, MA) was placed into the trachea and secured with 3–0 braided silk. Mice were transferred to a MiniVENT (Type 845, Harvard apparatus, Holliston, MA) for initiation of mechanical ventilation. Mice (n = 5 per group) were assigned to low tidal volume (LTV, 8 ml/kg) or high tidal volume (HTV, 15ml/kg) groups with either 0 or 4 cmH_2_O positive end-expiratory pressure (PEEP) and recruitment maneuvers (RM, inspiratory pressure of 20 cmH_2_O for 10 seconds every 20 minutes) or no RM. Respiratory rate was set at 100 bpm and FiO_2_ set at 40% for all groups. Mice were placed on warming pads and monitored continuously with assessment of heart rate, respiratory rate, SpO_2_, and response to noxious stimulation measured every 20 minutes. Lack of response to noxious stimuli was determined by a negative toe pinch response.

#### Two hour mechanical ventilation

Mice were assigned to low-tidal volume ventilation (LTV, 8 ml/kg), with PEEP (4cmH_2_O), with or without recruitment maneuvers versus high-tidal volume ventilation (HTV, 15 ml/kg) with ZEEP (0cmH_2_O), with or without recruitment maneuvers with 10 mice in each group including an air control group.

#### Two versus four hour mechanical ventilation

Mice were assigned to low-tidal volume ventilation (LTV, 8 ml/kg), with PEEP (4cmH_2_O), with recruitment maneuvers versus high-tidal volume ventilation (HTV, 15 ml/kg) with ZEEP (0cmH_2_O), with recruitment maneuvers with 5 mice in each group including an air control group.

### Measurements

Dynamic lung compliance (Cdyn) and resistance (Rrs) were measured with a plethysmograph for restrained animals (Buxco, Inc.). Arterial blood samples were collected prior to discontinuation of mechanical ventilation via the carotid artery. Samples were analyzed using a standard blood gas analyzer (ABL 815; Radiometer) within 15 minutes of sampling. Mice were euthanized at the end of the study period with an intraperitoneal overdose of pentobarbital. Lungs were lavaged twice with 1-ml aliquots of phosphate-buffered saline (PBS), pH 7.6. Collected bronchoalveolar lavage fluid was centrifuged in a benchtop unit at 2000 rpm for 10 minutes and the resulting pellet was suspended in PBS with a final volume of 500 μl and 120 μl of BAL fluid was used to prepare slides for total and differential cell counts. Supernatants were collected for cytokine levels (Milliplex; Millipore corp., St. Charles, Missouri, USA), total protein, and albumin (Micro BCA Protein Assay, Thermo Scientific, Rockford, IL, USA) analyses. Cytokines evaluated were IL-6, IL-1β, IL-10, KC, MIP-2, TNF-α, TGF-β. Diff-Quick (International Reagent Corp, Kobe, Japan) was used to stain slides for cytological analysis. The left lung was fixed for histopathologic analysis at 30cmH_2_O pressure with 1% paraformaldehyde and processed in paraffin for histology sampling.

#### Tissue staining and acute lung injury histology score

After fixation, the left lung was placed in 70% ethanol and prepared for paraffin embedding. Lung sections of 5-μm thickness were made with sectioning through the larger lobar bronchi in parallel. Sections were then dried and prepared for staining with hematoxylin and eosin for histology. Light microscopy was the primary technique used for slide analysis. Three independent, blinded observers used the acute lung injury scoring system recommended by the American Thoracic Society to assign a histology score to each slide (see Table 5).[16]

**Table 5:** Lung injury scoring system[34].

Parameter	0	1	2
(A) neutrophils in alveolar space	None	1–5	>5
(B) neutrophils in interstitial space	None	1–5	>5
(C) hyaline membranes	None	1	>1
(D) proteinaceous debris filling the airspaces	None	1	>1
(E) alveolar septal thickening	None	2x-4x	>4x

Score = [(20 x A) + (14 x B) + (7 x C) + (7 x D) + (2 x E)] / (number of fields x 100)

**score at least 20 random high-power fields (400x total magnification) **

### Definition of acute lung injury

As described previously in an official workshop at the American Thoracic Society (ATS) acute lung injury was defined as histological evidence of tissue injury, alteration of the alveolar-capillary barrier, presence of an inflammatory response, and evidence of physiologic dysfunction with three out of four of these criteria satisfying the definition of acute lung injury.[16] Based on this definition, our study was structured to evaluate histological evidence of tissue injury (lung injury scoring system), alteration of the alveolar capillary barrier (increase in BAL total protein and/or albumin), presence of an inflammatory response (increase in BAL absolute neutrophils and/or increase in proinflammatory cytokines in BAL fluid), and evidence of physiologic dysfunction (hypoxemia, PaO_2_/FiO_2_ < 200).

### Statistical analysis of data

GraphPad Prism 6 (Graphpad Software, San Diego, CA) was used for data analysis. D’Agostino-Pearson omnibus normality test was used to assess for normality in groups. Nonparametric data analysis included Kruskal-Wallis test with Dunn’s multiple comparison tests when possible; if not then a Mann-Whitney test (two-tailed, unpaired) was used and a Bonferroni correction was applied to test for statistical significance. Data was expressed in figures and texts as median ± the interquartile range. A prespecified p-value of < 0.05 was considered statistically significant.

## Results

### Two hour ventilation

#### Histology

**Fig 1:**
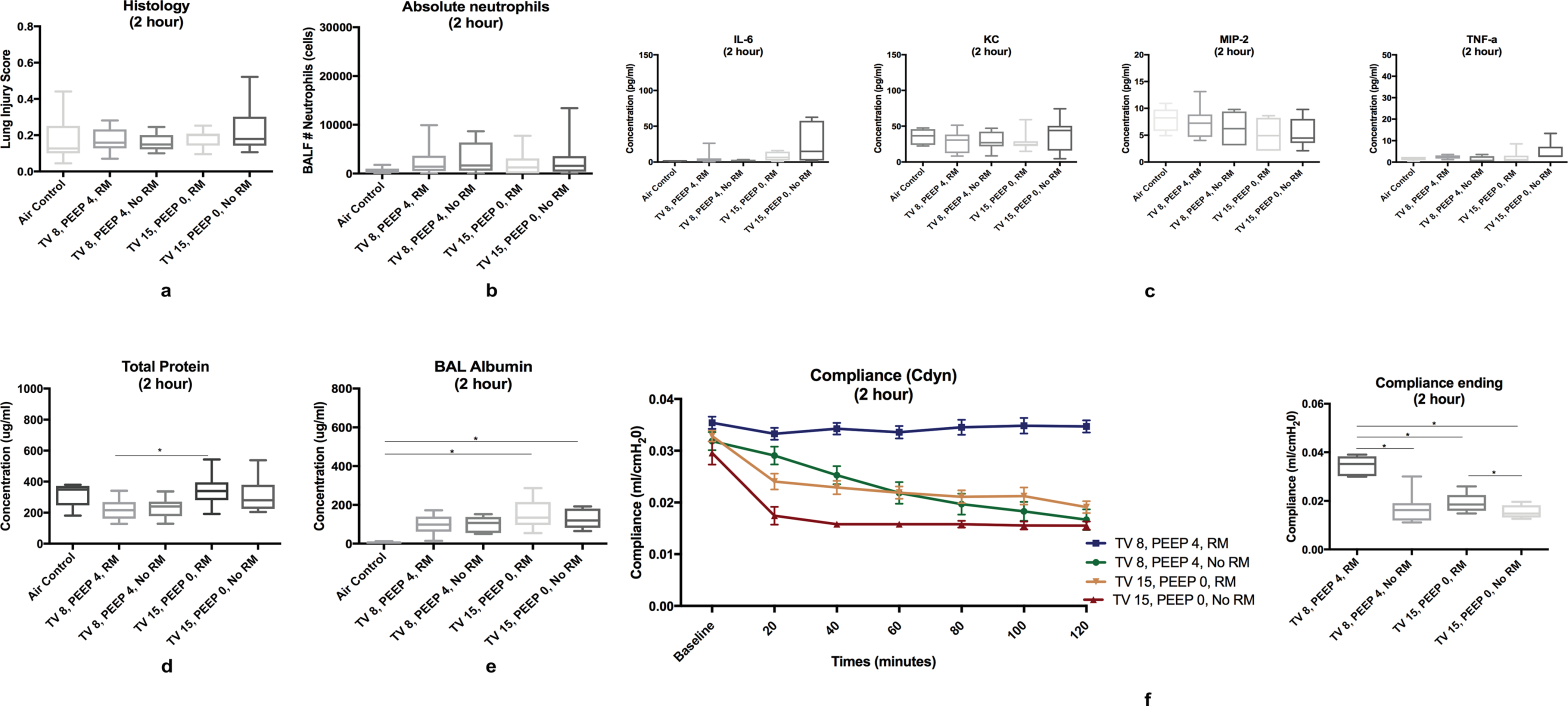
Positive-pressure ventilation (2-hour). **1a** No significant differences in histology lung injury scores. **1b** No significant differences were noted in bronchoalveolar absolute neutrophil counts. **1c** No significant differences were noted in bronchoalveolar lavage proinflammatory cytokines between all groups. 1**d** Bronchoalveolar lavage total protein was significantly elevated in the HTV RM group compared to the LTV RM group (p = 0.0086). **1e** Bronchoalveolar lavage albumin was significantly elevated in the high tidal volume groups compared to the air control group, but not when compared to the low-tidal volume ventilation groups (p = 0.0077) **1f** Pulmonary compliance was significantly higher in the low-tidal volume ventilation group (TV 8, PEEP 4, RM) compared to all other groups (p<0.0001) and in both LTV and HTV groups with RM versus no RM (p<0.0001, p = 0.0155). Data in boxplots is presented in median ± minimum and maximum values.

Histological measurements of tissue injury showed no significant differences in scores between the HTV (TV 15, PEEP 0, RM), LTV (TV 8, PEEP 4, RM), or air control groups (Fig 1A).

#### BAL cell counts and cytokine assay

No statistically significant differences in bronchoalveolar lavage absolute neutrophil counts were noted (Fig 1B); however, neutrophil counts were increased in all groups compared to the air group. No significant differences were noted in either proinflammatory or anti-inflammatory cytokines from bronchoalveolar lavage fluid (Fig 1C).

#### BAL total protein and albumin assays

Significant elevations of bronchoalveolar lavage total protein were noted in the HTV ventilation group (TV 15, PEEP 0, RM) compared to the LTV ventilation group (TV 8, PEEP 4, RM) (p = 0.0086) (Fig 1D). Bronchoalveolar lavage albumin was significantly elevated in the HTV groups compared to the air control group (p = 0.0077), but was not significantly elevated when the high tidal volume groups were compared to the low tidal volume groups (Fig 1E).

#### Pulmonary function

Measurements of physiology dysfunction did not show significant differences in partial pressure of arterial oxygenation (PaO_2_) or partial pressure of arterial oxygen to fraction of inspired oxygen (PaO_2_/FiO_2_) ratio (Fig 1F). Pulmonary compliance was significantly higher in the low-tidal volume ventilation group (TV 8, PEEP 4, RM) compared to all other groups (p<0.0001). Compliance was also significantly higher in the both the low and high tidal volume groups with RM than without RM (p<0.0001, p = 0.0155) (Fig 1F). Pulmonary resistance in the LTV group was significantly lower compared to all other groups (p<0.0001).

### Comparison of 2 hour ventilation to 4 hour ventilation

#### Histology

**Fig 2:**
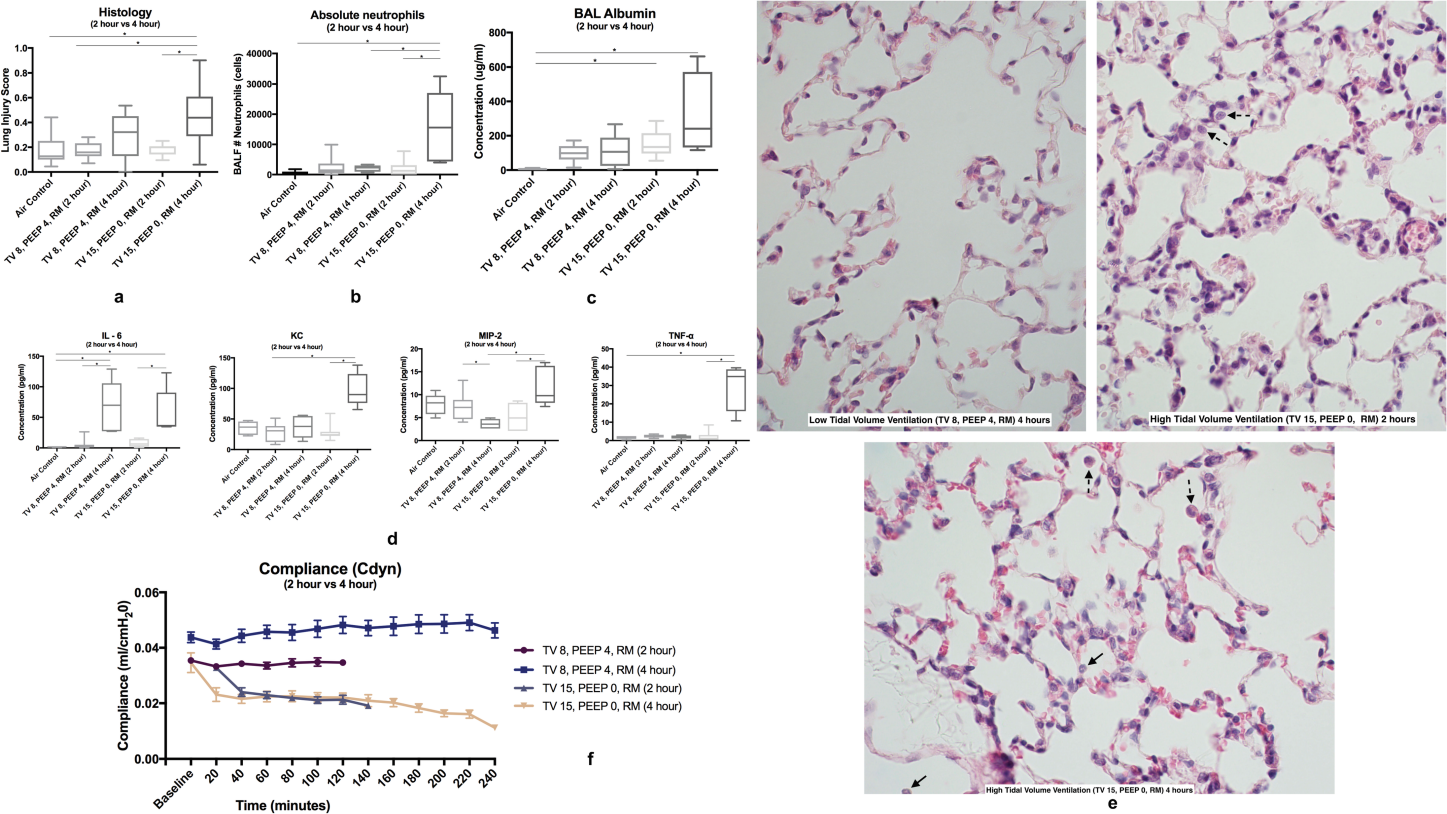
**Positive-pressure ventilation (2-hour vs 4-hour) 2a** Standardized histology scores were significantly higher in the HTV group at 4 hours compared to the air control, LTV group at 2 hours, and HTV group at 2 hours (p = 0.0002) **2b** Absolute neutrophils were significantly elevated in the TV 15, PEEP 0, RM group at 4 hours, but not at 2 hours of positive-pressure ventilation (p = 0.0046) and were significantly elevated in the HTV group compared to the LTV group (p = 0.0079) **2c** Bronchoalveolar lavage albumin was significantly increased in the HTV groups at 2 and 4 hours compared to the air control (p = 0.0046) **2d** Proinflammatory cytokines were significantly elevated in the HTV group at 4 hours, but not at 2 hours (see text for p values) **2e** Lung histology image stained with H&E. Top Left: LTV ventilation (TV 8, PEEP 4, RM (4 hour). Top Right: HTV ventilation (TV 15, PEEP 0, RM (2 hour)). Bottom: HTV ventilation (TV 15, PEEP 0, RM (4 hour)) **2f** Compliance was significantly higher in the LTV group at 4 hours compared to 2 hours of ventilation (p = 0.0027) and was significantly higher in the HTV group at 2 hours compared to 4 hours of ventilation (p = 0.0007). Data in boxplots is presented in median ± minimum and maximum values. *Neutrophils are denoted with an arrow*, *macrophages with a dotted line arrow*, *and proteinaceous debris with a curved line arrow*.

Standardized histology scores were significantly higher in the HTV group at 4 hours compared to the air control, LTV group at 2 hours, and HTV group at 2 hours (p = 0.0002) (Fig 2A). Select images of lung histology are shown in Fig 2E.

#### BAL cell counts and cytokine assay

Absolute neutrophils were significantly elevated in the high-tidal volume ventilation group (TV 15, PEEP 0, RM) at 4 hours (p = 0.0046), but not at 2 hours of positive-pressure ventilation and were significantly elevated in the high-tidal volume ventilation group compared to the low-tidal volume ventilation group (p = 0.0079) (Fig 2B). Bronchoalveolar lavage cytokines varied in both the HTV and LTV groups. IL-6 was significantly elevated in both the low and high tidal volume groups at 4 hours (p = 0.0006), but not at 2 hours of ventilation compared to the air group. IL-6 was also significantly elevated at 4 hours of ventilation compared to 2 hours of ventilation in both the LTV and HTV groups (p = 0.0025, p = 0.0043). TNF-α was significantly elevated in the HTV group compared to the air control at 4 hours of ventilation (p = 0.011), but not at 2 hours of ventilation (Fig 2D). A significant elevation was noted in the HTV groups between 2 hours and 4 hours of ventilation (p<0.025). KC was significantly elevated in the HTV group at 4 hours of ventilation compared to the LTV group at 2 hours of ventilation (p = 0.01). In the HTV group, KC was significantly increased after 2 additional hours of ventilation (p<0.025) (Fig 2D). MIP-2 was significantly elevated when the HTV group was compared to the LTV group at 4 hours of ventilation (p = 0.0067) and significant differences were noted in both the LTV and HTV group at 2 and 4 hours of ventilation (p = 0.0195 & 0.0088, respectively).

#### BAL total protein and albumin assays

Bronchoalveolar lavage total protein was significantly elevated in the HTV group at 2 hours compared to the LTV group at 4 hours of ventilation (p = 0.0038). Bronchoalveolar lavage albumin was significantly increased in the HTV groups at 2 and 4 hours compared to the air control (p = 0.0046) (Fig 2C). Bronchoalveolar lavage total cell counts were significantly increased in both HTV ventilation groups compared to the air group (p<0.0001).

#### Pulmonary function

Measurements of physiology dysfunction resulted in no significant differences in partial pressure of arterial oxygenation (PaO_2_) or partial pressure of arterial oxygen to fraction of inspired oxygen (PaO_2_/FiO_2_) ratios. Pulmonary compliance was significantly higher in the LTV groups compared to the HTV groups (p<0.0001). Compliance was significantly higher in the LTV group at 4 hours compared to 2 hours of ventilation (p = 0.0027) and was significantly higher in the HTV group at 2 hours compared to 4 hours of ventilation (p = 0.0007) (Fig 2F). Pulmonary resistance was significantly lower in the LTV groups compared to the HTV groups at both 2 hours and 4 hours of ventilation (p<0.0001). Resistance was significantly lower at 4 hours of ventilation compared to 2 hours of ventilation in the LTV group, but no significant differences were noted in the HTV group between 2 hours and 4 hours of ventilation.

### 4 hour ventilation with RM +/- PEEP

#### Histology

**Fig 3:**
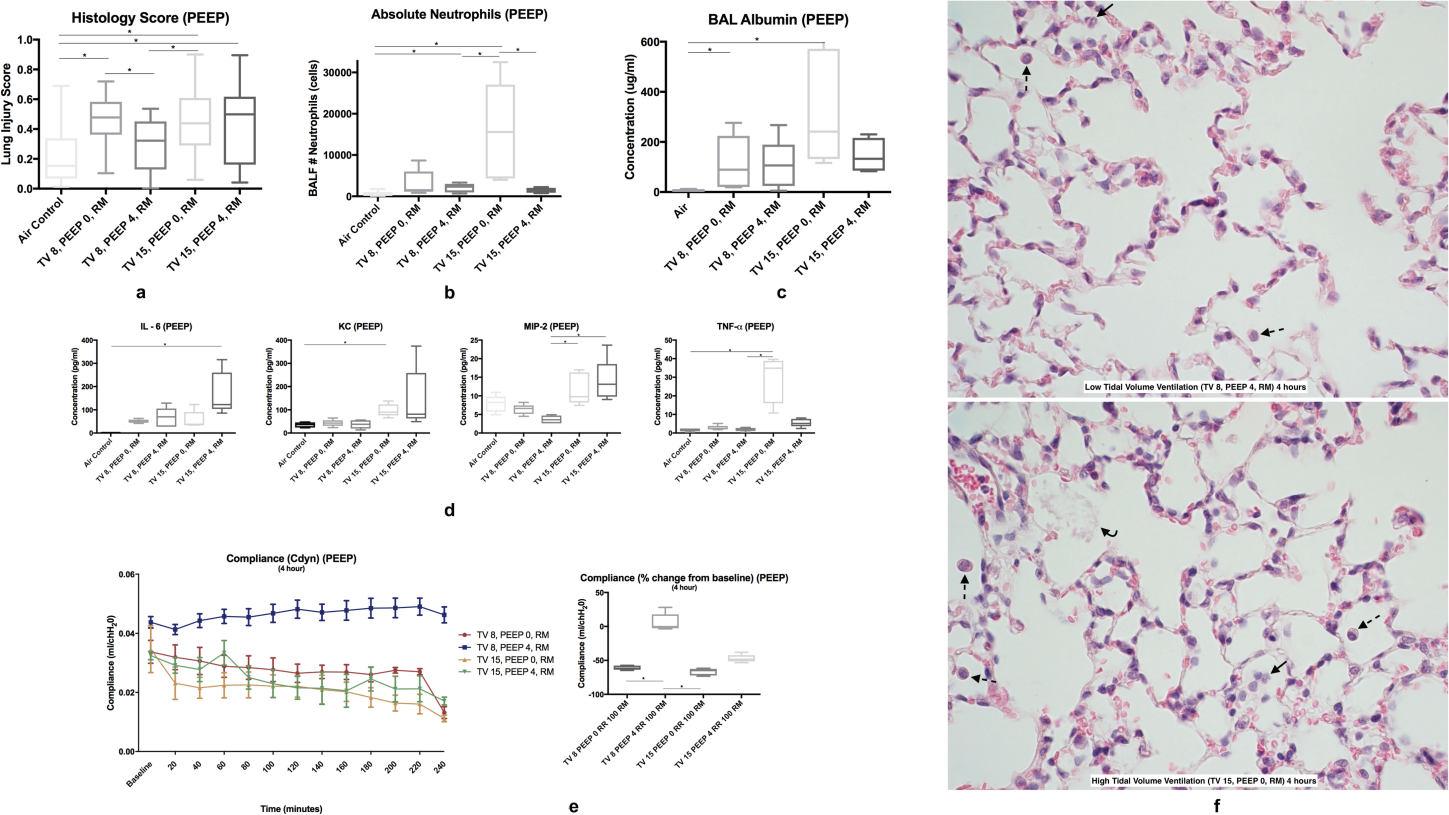
Positive-pressure ventilation (4-hour with RM +/- PEEP). **3a** Standardized histology scores were higher in all groups compared to the air control group, but not the TV 8, PEEP 4, RM group. The addition of PEEP in the LTV group resulted in a significant decrease in the histology score. **3b** Absolute neutrophils were significantly elevated in the TV 15, PEEP 0, RM group compared to the air control, TV 8, PEEP 4, RM, the TV 15, PEEP 4, RM group. The addition of PEEP to the HTV group resulted in a significant decrease in absolute neutrophils. **3c** BAL albumin was significantly increased in both LTV and HTV groups without PEEP compared to the air control group **3d** BAL cytokines were elevated in the HTV ventilation group. **3e** Pulmonary compliance in the LTV ventilation group was significantly higher compared to all other groups. **3f Left:** Lung histology image stained with H&E of low tidal volume (TV 8, PEEP 4, RM) showing influx of macrophages within the airway. **Right:** Lung histology image stained with H&E of high tidal (TV 15, PEEP 0, RM) showing neutrophils, proteinaceous debris, and hemorrhage within the airway. *Neutrophils are denoted with an arrow*, *macrophages with a dotted line arrow*, *and proteinaceous debris with a curved line arrow*.

Measurements of histological evidence of tissue injury showed that all groups had higher injury scores compared to the air control group, but the low-tidal volume ventilation group (TV 8, PEEP 4, RM) was not significantly elevated (Fig 3A). Significant elevations compared to the air group were noted in the TV 8, PEEP 0, RM group (p < 0.0001), TV 15, PEEP 0, RM group (p = 0.0005), and the TV 15, PEEP 4, RM group (p = 0.0019). The addition of PEEP in the LTV group resulted in a significant decrease in the histology score (p = 0.0145). Select images of lung histology are shown in Fig 3F.

#### BAL absolute neutrophil counts and cytokine assay

Bronchoalveolar lavage absolute neutrophils counts were significantly increased in the TV 15, PEEP 0, RM group compared to the air control (p<0.001), TV 8, PEEP 4, RM group (p = 0.0079), and the TV 15, PEEP 4, RM group (p = 0.0079). The addition of PEEP to the HTV group resulted in a significant decrease in absolute neutrophils (p = 0.0079) (Fig 3B). Bronchoalveolar lavage cytokines IL-6 and KC were significantly elevated in the TV 15, PEEP 4, RM group compared to the air control group (p = 0.0047, p = 0.0026). IL-10 was not significantly different amongst any group. TNF-α was significantly elevated in the TV 15, PEEP 0, RM group compared to both the air control and TV 8, PEEP 4, RM group (p = 0.0015) (Fig 3D). MIP-2 was significantly elevated in both HTV groups (TV 15, PEEP 0, RM and TV 15, PEEP 4, RM) compared to the LTV group (TV 8, PEEP 4, RM) (p = 0.0010).

#### BAL total protein and albumin assays

Measurements of alteration of the alveolar capillary barrier revealed elevation in bronchoalveolar lavage total protein in the TV 15, PEEP 0, RM group compared to the air group (p = 0.0159). Bronchoalveolar lavage albumin was significantly elevated in the TV 8, PEEP 0, RM (p = 0.0159) and the TV 15, PEEP 0, RM group (p = 0.018) when compared to the air control group (Fig 3C).

#### Pulmonary function

PaO_2_ or PaO_2_/FiO_2_ were significantly decreased in the LTV PEEP 0 group compared to the LTV PEEP 4 group (p = 0.0054). Pulmonary compliance in the TV 8, PEEP 4, RM was significantly higher compared to all other groups (p<0.0001). Ending compliance was significantly higher in the TV 8, PEEP 4, RM group compared to the TV 8, PEEP 0, RM group and TV 15, PEEP 0, RM group (p = 0.001) (Fig 3E). Pulmonary resistance was significantly lower in the TV 8, PEEP 4, RM compared to all other groups (p<0.0001). Ending resistance was significantly lower in the TV 8, PEEP 4, RM group compared to both the LTV and HTV groups without PEEP (TV 8, PEEP 0, RM and TV 15, PEEP 0, RM; p = 0.0008).

### 4 hour ventilation +/- PEEP and +/- RM

#### Histology

**Fig 4:**
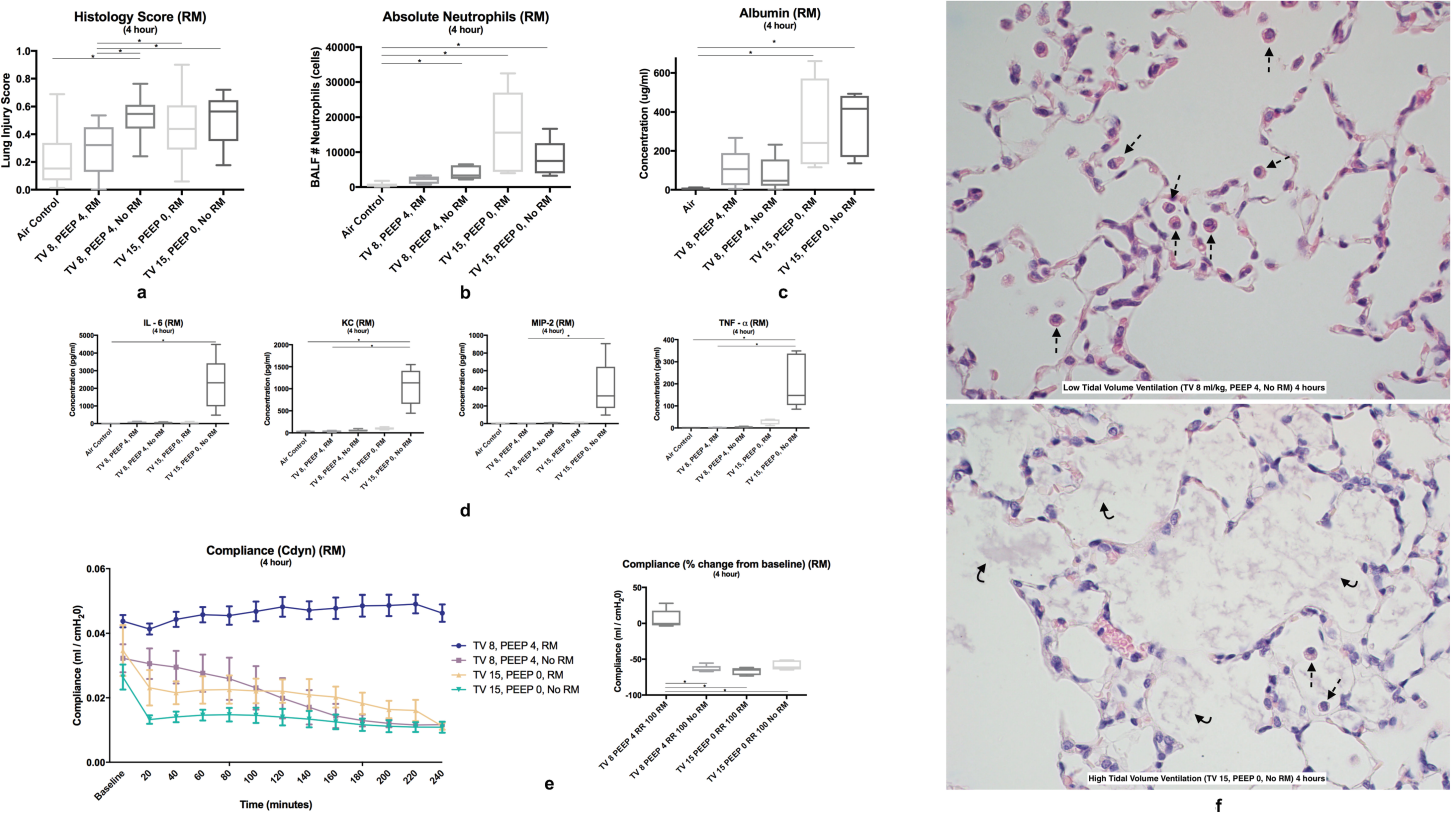
Positive-pressure ventilation (4-hour +/- PEEP and +/- RM). **4a** Standardized histology scores were increased in all groups except the low-tidal volume ventilation group (TV 8, PEEP 4, RM) compared to the air control group (not shown) and the LTV RM group. **4b** Bronchoalveolar lavage absolute neutrophil counts were significantly increased in the LTV group with no RM and both HTV groups compared to the air control group. **4c** Bronchoalveolar lavage albumin was significantly increased in both HTV groups compared to the air control group. **4d** Proinflammatory cytokines were significantly increased in the HTV group with no RM. **4e** Pulmonary compliance was significantly elevated in the LTV ventilation group compared to all other groups. Lung histology sections are shown from all groups. **4f Left:** Lung histology section stained with H&E of LTV with no RM showing macrophages within the airway. **Right:** Lung histology section stained with H&E of HTV with no RM showing proteinaceous debris within the airway. *Neutrophils are denoted with an arrow*, *macrophages with a dotted line arrow*, *and proteinaceous debris with a curved line arrow*.

Standardized histology scores were significantly elevated in the TV 8, PEEP 4, No RM group and both HTV groups (TV 15, PEEP 0, RM and TV 15, PEEP 0, No RM), but not the TV 8, PEEP 4, RM group (p<0.0001) (Fig 4A). In the LTV groups, the addition of RM significantly reduced the histology score (p = 0.0007). Select images of lung histology are shown in Fig 4F.

#### BAL absolute neutrophil counts and cytokine assay

Bronchoalveolar lavage absolute neutrophils counts were significantly increased in the LTV group with no RM (TV 8, PEEP 4, No RM) and both HTV groups (TV 15, PEEP 0, RM and TV 15, PEEP 0, No RM) (p<0.0001) (Fig 4B). Bronchoalveolar lavage cytokines including IL-6 were significantly elevated in the TV 15, PEEP 0, No RM group compared to the air control (p = 0.0018). IL-10 showed no significant differences in groups. TNF-α and KC were significantly elevated in the TV 15, PEEP 0, No RM group compared to both the air control and TV 8, PEEP 4, RM groups (p = 0.0004, p = 0.001) (Fig 4D). MIP-2 was significantly elevated in the TV 15, PEEP 0, No RM groups compared to the TV 8, PEEP 4, RM group (p = 0.0006).

#### BAL total protein and albumin assays

Total protein was significantly elevated in both HTV groups (TV 15, PEEP 0, RM and TV 15, PEEP 0, No RM) compared to the air control (p = 0.0159, p = 0.0079). Total protein was significantly elevated in the TV 15, PEEP 0, No RM group compared to the TV 8, PEEP 4, RM group. Albumin was significantly increased in both HTV groups (TV 15, PEEP 0, RM and TV 15, PEEP 0, No RM) compared to the air control (p<0.0053) (Fig 4C).

#### Pulmonary function

No significant differences were noted in PaO_2_ or PaO_2_/FiO_2_ amongst all groups. Pulmonary compliance was elevated in the TV 8, PEEP 4, RM group compared to all other groups (p<0.0001) (Fig 4E). Addition of RM in both the LTV and HTV groups resulted in improved compliance (p<0.0001, p<0.0006). Ending compliance was higher in the TV 8, PEEP 4, RM compared to both HTV groups (p = 0.0099). Pulmonary resistance was higher in all groups compared to the TV 8, PEEP 4, RM group (p<0.0001). Ending resistance was lower in the TV 8, PEEP 4, RM group compared to both HTV groups (p = 0.0024) in addition to the LTV group with no RM (TV 8, PEEP 4, No RM) (p = 0.0079).

## Discussion

Use of low tidal volumes and positive end-expiratory pressure are essential components of ARDS ventilation management, yet even low-tidal volume ventilation strategies can result in VILI. While volutrauma and atelectrauma have been identified as the main clinically modifiable drivers of VILI, off-target delivery of mechanical ventilation remains common and the thresholds for exposure to injurious ventilation settings both in terms of magnitude and duration of exposure remain incompletely understood. Using clinically relevant ventilation strategies, we used a one-hit model for VILI to determine the early time points in the development of VILI and evaluate the application of PEEP and RM as independent factors.

Previous studies have noted no differences in the lung inflammatory response between low tidal volume and high tidal volume groups undergoing short term ventilation (one-hour of ventilation).[24,25,26] At 2 hours of high-tidal volume ventilation there was no evidence of histological tissue injury, inflammatory response, or physiologic dysfunction; although there was evidence of alteration in the alveolar-capillary barrier and pulmonary function testing. Bronchoalveolar lavage fluid albumin was increased in the high tidal volume group compared to the air controls, but a significant difference was not noted between the low- and high-tidal volume groups. Alterations of the alveolocapillary barrier is a critical feature that defines acute lung injury in both humans and animals. These alterations result from damage to the endothelium and formation of intercellular gaps, which led to increased microvascular permeability, decreased fluid transport, and lack of surfactant production.[16,27,28] Both LTV and HTV in short-term ventilation caused influx of alveolar proteinaceous debris, yet no differences were noted between the groups, indicating that short term ventilation results in minimal influx of proteinaceous debris into the alveolar space. Significant influx is noted at 4 hours, indicating a time component to development of pulmonary edema associated with VILI in these experimental groups. Neutrophil recruitment into the interstitium and bronchoalveolar space occurs after damage to the endothelium and with elevation in chemokines and cytokines.[29] Based on our results, initial damage to the endothelium occurs within 2 hours of ventilation, as evident by the influx of proteinaceous debris; whereas the influx of neutrophils into the alveolar space requires more than 2 hours of ventilation at HTV and ZEEP. Ventilation at both low-tidal and high-tidal volume resulted in minimal acute lung injury at 2 hours of ventilation, yet significant elevations in histology injury score, absolute neutrophil influx into the alveoli, and pro-inflammatory cytokines occurred at 4 hours of ventilation indicating a significant increase in lung injury at the 4 hour time point compared to 2 hours of ventilation. Significant lung injury occurs at 4 hours of ventilation, but not at 2 hours of ventilation in this study. Future studies are indicated to assess for reversibility of lung injury in non-terminal studies with short- and long-term ventilation.

Proinflammatory cytokines (IL-6, TNF-α, IL1-β, IL-8 [KC, MIP-2]) and anti-inflammatory cytokines (TGF-β, IL-10) were evaluated in this study to evaluate the inflammatory response in bronchoalveolar lavage fluid. Acute lung injury is typically associated with increases in both proinflammatory and anti-inflammatory cytokines. IL-8 is considered a prominent neutrophil-recruiting chemokine; the murine functional orthologues are KC (CXCL1) and MIP-2 (CXCL2).[30] In studies that have evaluated human patients with ARDS, BAL concentrations of many proinflammatory cytokines are increased in patients with ARDS and were reduced in patients with low-tidal volume ventilation compared to traditional ventilation.[5,31,32] In studies that have evaluated ventilator-induced lung injury in mice, proinflammatory cytokines are upregulated in VILI.[23,33,34,35] Much less is known about the early molecular events in clinically relevant mouse VILI models because they frequently involve exposure to supraphysiologic high-tidal volume ventilator settings, making it difficult to extrapolate these findings to human patients.[21,23,36] Biotrauma is not expected to occur at the initial stages of ventilator-induced lung injury as shown with minimal elevation at 2 hours, but significant elevation occurred at 4 hours (IL-6, KC, TNF-α). IL6, KC, MIP-2, and TNF-α were highest in the HTV, ZEEP, no RM group compared to all other groups, indicating a significant protective effect of RM even in groups with significant volutrauma. Atelectrauma appears to be a stronger stimulus for cytokine recruitment compared to volutrauma and the use of recruitment maneuvers can significantly mitigate atelectrauma.

Few human studies have addressed the role of early use of low-tidal volume ventilation in determining clinical outcomes. Only two observational studies have investigated the association between early LTV and survival in patients with ARDS.[12,37] Checkley et al studied a cohort of approximately 2100 patients enrolled in ARDSnet clinical trials within 36–48 hours of the onset of ARDS from 1996–2005 and subsequently managed with LTV, and assessed for an association between pre-enrollment TV and hospital mortality.[37] The authors found no association between TV in the 48 hours before ARDS onset and mortality in multivariable analysis adjusted for multiple potential confounders. In contrast, Needham et al examined a cohort of 482 subjects ventilated for 5 days with new-onset ARDS prospectively enrolled from 2004–2007 and found a significant association between initial use of LTV (defined at ≤ 6.5 ml/kg of predicted body weight) and ICU mortality.[12] On adjusted analysis, the authors found a 23% increase in the risk of ICU mortality for every 1 ml/kg of patient body weight increase in initial TV after the onset of ARDS. In addition to these studies, several other lines of clinical evidence suggest that early use of low-tidal volume ventilation strategies may improve patient outcomes including reduced perioperative pulmonary complications in patients receiving low-tidal volume ventilation intraoperatively and lower rates of hospital-acquired pulmonary complications including new ARDS in patients without existing ARDS who receive low-tidal volume ventilation in the first 24 hours after intubation.[38,39,40] We recognize that mice differ from humans in the inflammatory response to lung injury including lack of hyaline membrane formation, fewer circulating neutrophils (10–25% versus 50–70%), lack of defensins, differences in cytokine profiles, and lack of co-morbidities.[16,41] Despite these differences, our findings support a potential role for pro-inflammatory cytokines in early phases of VILI. Additional research is still needed into the relative contributions of pro- and anti-inflammatory pathways to the early pathogenesis of VILI, both in humans without existing lung injury and in animal models under a variety of low- and high-tidal volume ventilation.

Pulmonary compliance decreases in acute lung injury due to pulmonary edema, atelectasis, and cellular infiltration, as well as de-recruitment of alveoli, have been associated with reduced oxygenation.[42,43] Recruitment maneuvers are used to reopen unstable airless alveoli through a transient increase in transpulmonary pressure with the intent to improve gas exchange and prevent atelectrauma.[44] Recruitment maneuvers in the use mechanical ventilation are controversial due to a lack of standardization as well as contradictory results regarding clinical benefits and the risk of hemodynamic compromise.[42,44,45,46,47,48,49,50] Recent reviews of recruitment maneuvers for acute lung injury have concluded that routine clinical use cannot be recommended or discouraged due to insufficient evidence. However, recruitment maneuvers can be considered for patients with acute lung injury and life-threatening hypoxemia due to their transient effects on hypotension and desaturation as applied during this study as recruitment maneuvers were essential to maintaining dynamic compliance.[44,51]

Recruitment of alveoli occurs at different pressures throughout the lung parenchyma with the most recruitable lung opening after two seconds of airway pressure.[14] Recruitment maneuver techniques are varied throughout the literature and in practice. In animal studies, common values are from 20 cmH_2_O to 40cmH_2_O with all of the referenced studies emploring a 1-hit model except for Allen et al [52] using a 2-hit model of lung injury.[52,53,54,55,56,57,58] Zosky et al (2008) determined that pressure greater than 20 cmH_2_O result in increased compliance.[58] Reiss et al (2011) determined that the use of frequent low level RM (30 cmH_2_O for 1 second every 5 minutes) was protective and lung injury was noted when the duration of RM was limited to once every hour.[57] Cannizzaro et al (2009) determined that low volume RM (25 cmH_2_O every 5 or 75 minutes) did not result in stable pulmonary mechanics compared to high volume RM (35 cmH_2_O).[54] Whereas, Allen et al (2004) determined that RM (25cmH_2_O) resulted in stable pulmonary mechanics in non-injured lungs; however, this RM may have been inadequate for injured lungs (acid-induced lung injury) and RM with every breath is injurious in high tidal volume ventilation, but not in low tidal volume ventilation, suggesting that further studies are required to determine the optimal frequency of RM at a time point between 1 minute and 1 hour.[52,53] Low volume recruitment (8 cmH_2_O for 10 breaths, every 30 minutes) resulted in improved pulmonary mechanics compared to no recruitment.[55] Ko et al (2008) applied RM at 40 cmH_2_O for 10 seconds every 15 minutes and noted improved oxygenation, pulmonary mechanics, and attenuation of inflammation with the additive effect of PEEP.[56] Frank et al (2005) determined that the use of RM (two 30cmH_2_O RM separated by 1 minute every 30 minutes) resulted in sustained improvements in oxygenation, compliance and lung volume.[59] Application of low-volume RM for a short duration with a relatively moderate frequency is critical for minimizing lung injury in patients with atelectasis as noted in this study.

Based on our previous pilot studies, we determined that an opening pressure of 20cmH_2_O for 10 seconds with repetition every 20 minutes resulted in a consistent pulmonary compliance in the LTV group. RM improved pulmonary compliance in the HTV group; however, this did not reach statistical signifance and was only a mild improvement. RM reduced overall acute lung injury in both LTV and HTV groups, yet only the LTV group reached statistical significance. Pro-inflammatory cytokines showed the most significant difference with RM in the HTV group, with significant increases in IL-6, KC, MIP-2, and TNF-α without the use of RM compared to the use of RM, indicating that atelectrauma appears to be a more important driver of cytokine influx than volutrauma. Neutrophil influx appeared to be associated with both volutrauma and atelectrauma, with a higher trend with the use of RM, indicating that at 4 hours of ventilation, neutrophil influx appears to be more associated with volutrauma than atelectrauma. Further studies of ventilator-induced lung injury past 4 hours should be pursued to evaluate this trend. RM are protective and should be considered to help ameliorate ventilator-induced lung injury and use of dynamic compliance can be used guide the frequency of RM. It should be noted, however, that clinical studies of RM typically perform RM considerably less frequently than our protocol, such as daily or every 8 hours as well as using higher airway pressures and breath holds of longer duration.[45,60,61,62,63] This difference in timing may explain the unclear benefits of clinically performed RM compared to our data and future research addressing the impact of RM on VILI will need to address the relationship between the intensity, duration, and frequency of RMs, and signs of lung injury.

PEEP was protective in both low and high tidal volume groups, but unable to ameliorate lung injury as evident by alterations in pro-inflammatory cytokines. PEEP attenuated the inflammatory response in the high tidal volume group as determined by a reduction in the absolute neutrophil counts and bronchoalveolar lavage albumin; whereas, a significant reduction in the histology score was noted in the low tidal volume group. PEEP attenuated the damage to the alveolar-capillary barrier as evident by a reduction in albumin influx into the alveoli. Yet, even at low-tidal volume ventilation, elevations in pro-inflammatory cytokines occurred and resulted in significant elevations in HTV even when PEEP was added. As determined in previous studies, increasing the PEEP to 6 cmH_2_O resulted in improved or equivocal protective ventilation than the synergistic effects of PEEP at 2 cmH_2_O and RM, suggesting a way to reduce lung injury by minimizing atelectrauma.[52,53,54,56,57] Use of PEEP has been well-established as an aspect of protective ventilation, our use of ZEEP was applied to maximize the injury in the high-tidal volume group in this model. It can be speculated that atelectrauma appears to be a more significant contributor to ventilator-induced lung injury than volutrauma at physiological ranges of tidal volume, yet further research is indicated to evaluate this concept at supraphysiological tidal volumes.

Similar VILI models previously used supraphysiologic tidal volumes to reproduce injury, the tidal volumes used in our study were lower and more clinically relevant.[20,21,23] With this study, we also attained ventilator-induced lung injury without utilizing a second insult and evaluated temporal changes (early times points at initial injury and later timpoints with progression of VILI) in TV, PEEP, and RMs. It is an ideal system to study the mechanisms that lead to VILI, as well as develop preventative therapies, or identify optimum ventilation strategies to minimize, or altogether prevent, VILI.

## Limitations

Inherent limitations are present in this study due to the relatively small sample size in each of the groups, which may have resulted in type II errors. Gender bias was present in our study as only female mice were used. Protease inhibitors were not added to the bronchoalveolar lavage samples, which may have affected sample analysis, especially considering that cytokines are easily degraded. Due to the limitations of our ventilator system, we were unable to measure the peak inspiratory pressure, which would have aided in more direct comparison to other studies of ventilator-induced acute lung injury in a translational aspect. Hemodynamic compromise is one of the main limiting factors of the use of recruitment maneuvers, within our study we were unable to directly monitor direct arterial blood pressure and oxygenation levels during recruitment maneuvers. It can be hypothesized that in mice, a tidal volume of 8 ml/kg with a PEEP of 4cmH_2_O was not sufficient to minimize atelectasis; hence, the need for recruitment maneuvers to maintain pulmonary compliance. Patients with severe lung injury are ventilated with LTV to reduce lung injury, whereas our mice had no lung injury prior to initiation of PPV; therefore, a lower tidal volume may inadvertently be harmful due to reduced alveolar recruitment. Alveolar recruitment occurs during tidal inflation and with reduced tidal volumes in patients with healthy lungs, alveoli may remain collapsed or compressed, worsening gas exchange.[64]

## Conclusions

Short term ventilation using clinically relevant positive pressure ventilator settings for 2 hours resulted in biologically-evident ventilator-induced lung injury with increased influx of total protein and albumin within the alveoli. Increasing the duration of PPV up to 4 hours results in progressive injury with histological evidence of acute lung injury, alteration of the alveolar-capillary membrane, and evidence of an inflammatory response with influx of neutrophils and increased proinflammatory cytokines. Low tidal volumes, positive end-expiratory pressure, and frequent recruitment maneuvers each showed contributions to lung protection, yet mild ventilator-induced lung injury still occurred. Our findings highlight the importance of studying the influence of multiple clinically relevant, potentially protective ventilation strategies at early time points in the evolution of ventilator-induced lung injury. This strategy will help to elucidate novel pathways in the pathogenesis of VILI and lead to new treatments to prevent or ameliorate the adverse effects of mechanical ventilation for patients with acute respiratory failure.

## Supporting information

S1 Data Raw data is available in the attached supporting information. (XLSX)

## Abbreviations

SC: subcutaneous
IP: intraperitoneal
HTV: high tidal volume
LTV: low tidal volume
PEEP: positive end-expiratory pressure
ZEEP: zero positive end-expiratory pressure
SpO2: pulse oximetry
PaO2: partial pressure of arterial oxygen
RM: recruitment maneuvers
ATS: American Thoracic Society
IL-6: interleukin-6
IL-1β: interleukin-1beta
IL-10: interleukin-10
KC: keratinocyte chemoattractant, chemokine C-X-C motif ligand 1 (CXCL1)
MIP-2: macrophage inflammatory protein, C-X-C motif ligand 2 (CXCL2)
TNFα: tumor necrosis factor alpha
TGF-β: transforming growth factor beta 1
